# Fab-based inhibitors reveal ubiquitin independent functions for HIV Vif neutralization of APOBEC3 restriction factors

**DOI:** 10.1371/journal.ppat.1006830

**Published:** 2018-01-05

**Authors:** Jennifer M. Binning, Amber M. Smith, Judd F. Hultquist, Charles S. Craik, Nathalie Caretta Cartozo, Melody G. Campbell, Lily Burton, Florencia La Greca, Michael J. McGregor, Hai M. Ta, Koen Bartholomeeusen, B. Matija Peterlin, Nevan J. Krogan, Natalia Sevillano, Yifan Cheng, John D. Gross

**Affiliations:** 1 Department of Pharmaceutical Chemistry, University of California, San Francisco, California, United States of America; 2 Keck Advanced Microscopy Laboratory and Department of Biochemistry and Biophysics, University of California, San Francisco, California, United States of America; 3 Howard Hughes Medical Institute, University of California, San Francisco, California, United States of America; 4 J. David Gladstone Institutes, San Francisco, California, United States of America; 5 Department of Cellular and Molecular Pharmacology, University of California, San Francisco, California, United States of America; 6 California Institute for Quantitative Biosciences, QB3, University of California, San Francisco, San Francisco, California, United States of America; 7 Department of Medicine, University of California, San Francisco, California, United States of America; 8 Department of Microbiology, University of California, San Francisco, California, United States of America; 9 Department of Immunology, University of California, San Francisco, California, United States of America; University of Illinois at Chicago College of Medicine, UNITED STATES

## Abstract

The lentiviral protein Viral Infectivity Factor (Vif) counteracts the antiviral effects of host APOBEC3 (A3) proteins and contributes to persistent HIV infection. Vif targets A3 restriction factors for ubiquitination and proteasomal degradation by recruiting them to a multi-protein ubiquitin E3 ligase complex. Here, we describe a degradation-independent mechanism of Vif-mediated antagonism that was revealed through detailed structure-function studies of antibody antigen-binding fragments (Fabs) to the Vif complex. Two Fabs were found to inhibit Vif-mediated A3 neutralization through distinct mechanisms: shielding A3 from ubiquitin transfer and blocking Vif E3 assembly. Combined biochemical, cell biological and structural studies reveal that disruption of Vif E3 assembly inhibited A3 ubiquitination but was not sufficient to restore its packaging into viral particles and antiviral activity. These observations establish that Vif can neutralize A3 family members in a degradation-independent manner. Additionally, this work highlights the potential of Fabs as functional probes, and illuminates how Vif uses a multi-pronged approach involving both degradation dependent and independent mechanisms to suppress A3 innate immunity.

## Author summary

Restriction factors are cellular proteins that inhibit viral replication and represent a first line of defense against viral pathogens. The APOBEC3 (A3) family of restriction factors plays an important role in blocking retroviral infections. HIV-1 encodes the Vif protein to antagonize A3, which allows spread of virus in host and ultimately development of AIDS. Prior studies indicate that Vif hijacks host proteolysis pathways to degrade A3 restriction factors; however, our work demonstrates that Vif can neutralize A3s in a degradation-independent manner. These findings suggest viral suppressors of innate immunity work by multiple mechanisms to ensure robust replication. Knowledge of such mechanisms is critical for development of therapeutic strategies to restore the ability of the immune system to cripple viral infections.

## Introduction

Despite tremendous advances in control and prevention, HIV has remained a persistent worldwide health concern. Current anti-retroviral treatments often use a combination of drugs that target key viral enzymes such as the protease, integrase, and reverse transcriptase [1]. The existing treatment options have proven to be successful in controlling viral load, yet with long-term use drugs frequently become ineffective as the virus acquires escape mutations that confer resistance. As multidrug-resistant strains of HIV become more prevalent, it is imperative to identify novel drug targets and innovative treatment strategies to counteract HIV.

The HIV protein Viral Infectivity Factor (Vif) has become recognized as a promising therapeutic target [1–3]. The primary function of Vif is to counteract the antiviral effects of host APOBEC3 (A3) innate immune proteins. In the absence of Vif, A3 proteins are packaged into virions and restrict lentiviral replication by inducing hypermutation of the viral genome [4–7]. Humans encode seven A3 proteins (A, B, C, D, F, G, and H) of which A3D, A3F, A3G, and A3H are able to restrict HIV-1. Vif antagonizes A3 proteins by hijacking a cellular Cullin-RING ubiquitin ligase, resulting in their ubiqutination and subsequent proteasomal degradation [5,8–13]. Importantly, Vif is conserved in all existing lentiviruses and its adaptation allowed interspecies transmission from old-world monkeys to chimpanzees, leading to the AIDS pandemic. Thus, not only is Vif-APOBEC3 an antiretroviral drug target for restoration of a key defense mechanism of our innate immune system, the details of its interactions with APOBEC3 family members can provide insights into the molecular arms-race between lentiviruses and their hosts [1,14].

As Vif is required for viral replication both in cell culture and *in vivo*, inhibition of Vif is an attractive strategy to restore the restrictive function of A3 family members and in turn cripple HIV infection. We and others have demonstrated that pharmacological inhibition of Vif unleashes the restriction potential of A3 family members [15–20]; however, the targets of these studies are either unknown or are host proteins required for Vif function. Although these studies demonstrate the effectiveness of antagonizing Vif to counteract HIV, targeting host factors may result in undesirable pleiotropic, off-target effects. A number of challenges currently impede our ability to develop inhibitors that directly target Vif. First, therapeutically targeting non-enzymatic proteins, especially protein-protein interfaces (PPIs), has posed a major obstacle for drug development. Second, there is no available structure of a Vif-A3 complex, thus limiting our molecular understanding of the Vif-A3 interactions. Third, Vif engages multiple A3 proteins (A3D/A3F, A3G, and A3H) through distinct binding surfaces. Lastly, it is becoming apparent that Vif antagonizes A3 proteins through multiple mechanisms including ubiquitin-mediated proteolysis as well as downregulating A3 at the transcriptional level by modulating the cellular RUNX transcription pathway [21,22]. Therefore, it is important to have a comprehensive understanding of Vif-mediated A3 neutralization to develop therapeutic strategies that unleash the innate immunity provided by A3 family members.

**Fig 1:**
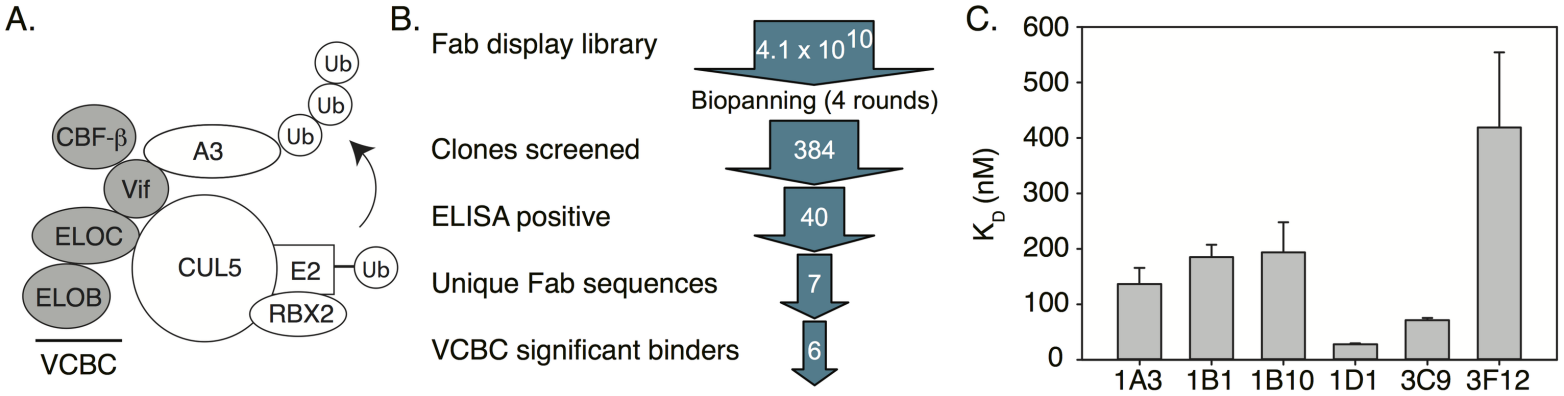
Fabs bind VCBC with high affinity. (A) Schematic representation of the Vif E3-ligase with the VCBC components denoted in gray. (B) Overview of Fab panning pipeline. (C) Binding affinities for VCBC-Fab interactions measured by BLI. Consensus Vif was obtained from the Los Alamos National Lab HIV Sequence Database (Alignment ID: 104CP14).

In an effort to develop tools to characterize the structure and function of Vif-A3 complexes, we have generated antibody antigen-binding fragments (Fabs) to the minimal Vif complex capable of binding A3 family members. This complex, termed VCBC, consists of HIV-1 Vif, the transcription co-factor CBFβ, and adapter proteins ELONGIN B and C (Fig 1A). Through a combination of enzymology, structural biology, and biochemistry, we have utilized the VCBC-binding Fabs to probe Vif functions and uncover novel mechanistic insight into Vif-mediated APOBEC3 restriction. Two Fabs were found to inhibit A3 ubiquitination by distinct mechanisms as one Fab restored A3 packaging and the other did not. Further investigation revealed that blocking VCBC binding to CULLIN-5 (CUL5) disrupted A3 ubiquitination as expected, but, surprisingly, was insufficient to restore A3 antiviral activity. These data demonstrate a degradation-independent mechanism for Vif-mediated A3 inhibition, further establishing that Vif uses a multi-pronged approach to antagonize A3 restriction factors. Additionally, these data show that pharmacological inhibition of A3 ubiquitination may be insufficient for a protective effect; therefore, guiding how we develop and screen future Vif inhibitors. Together, these findings highlight the utility of biologics as structural and functional probes, provide insight into strategies for inhibiting Vif, and establish a novel mechanism by which Vif can antagonize A3 proteins.

## Results

### Generation of Fabs against the A3 substrate receptor VCBC

We used a human naïve B cell phage library with a diversity of 4.1 x 10^10^ to identify Fabs against VCBC [23,24]. A VCBC substrate receptor complex containing consensus Vif was used as “bait” for Fab generation as it is able to form a functional E3 ligase and support viral infectivity in non-permissive cells, and displays 88.54% and 89.06% identity to HxB2 and NL4-3 Vifs, respectively, suggesting that isolated Fabs may be cross-reactive with multiple Vif complexes (S1 Fig). After four rounds of selection, we identified 6 VCBC-binding Fabs that bound to VCBC with dissociation constants (K_D_) in the low nanomolar range as determined by BioLayer Interferometry (BLI) (Fig 1, S2 Fig). To further characterize the Fabs, a competitive ELISA was used to assess whether they bound to overlapping epitopes. Fabs 1D1 and 3F12 did not compete with any other Fab, indicating they recognize unique epitopes on VCBC. Fabs 1A3, 1B3, 1B10, and 3C9 were all able to compete with one another for VCBC binding, suggesting that these Fabs bind to overlapping epitopes. 3C9 was the most potent in the competition assay, and was therefore chosen, along with 1D1 and 3F12, for further characterization.

### Fabs 3C9 and 1D1 inhibit Vif-mediated A3 ubiquitination

**Fig 2:**
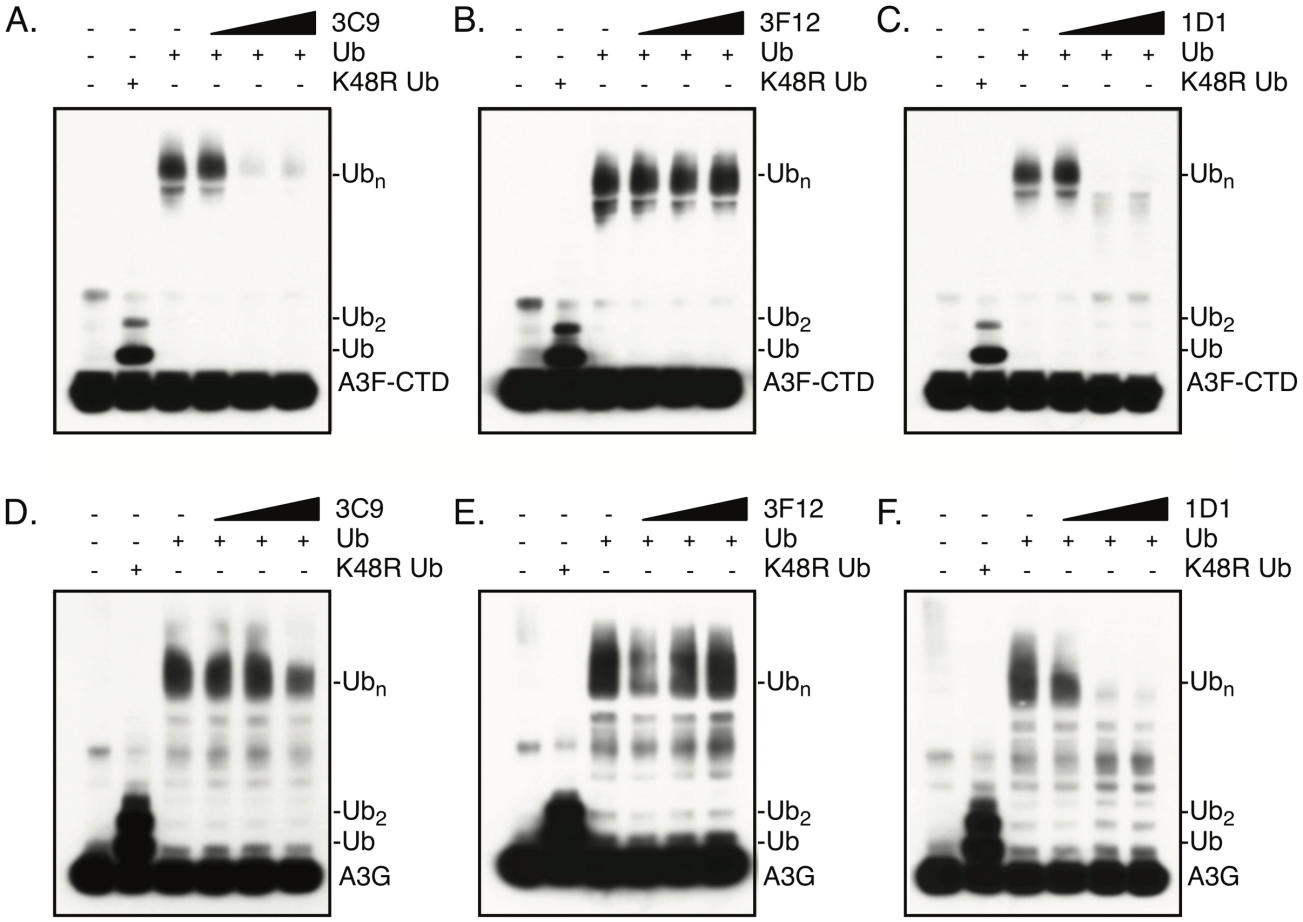
3C9 and 1D1 disrupt Vif-mediated A3 ubiquitination *in vitro*. Ubiquitination of Myc-tagged (A-C) A3F-CTD or (D-F) A3G in the absence or presence of increasing amounts of 3C9, 3F12, and 1D1 (0.1 μM, 0.5 μM, and 1μM). NL4-3 Vif E3 ligase was used for ubiquitination assays and A3 proteins were visualized by WB with anti-Myc.

We first categorized the Fabs based on their ability to disrupt Vif-mediated ubiquitination *in vitro* using either the Vif-binding, C-terminal domain of A3F (A3F-CTD) or full-length A3G (Fig 2). In the absence of Fabs, the NL4-3 Vif E3-ligase is able to poly-ubiquitinate A3F-CTD and A3G (Fig 2). Fabs 3C9, 1D1, and 3F12 were individually titrated into the ubiquitination assay, and we observed that 3C9 and 1D1 were able to disrupt Vif-mediated A3 ubiquitination in a dose dependent manner, whereas 3F12 had no effect (Fig 2). Specifically, 3C9 was able to inhibit poly-ubiquitination of A3F-CTD, but not A3G, and 1D1 was able to inhibit both A3F-CTD and A3G poly-ubiquitination.

**Fig 3:**
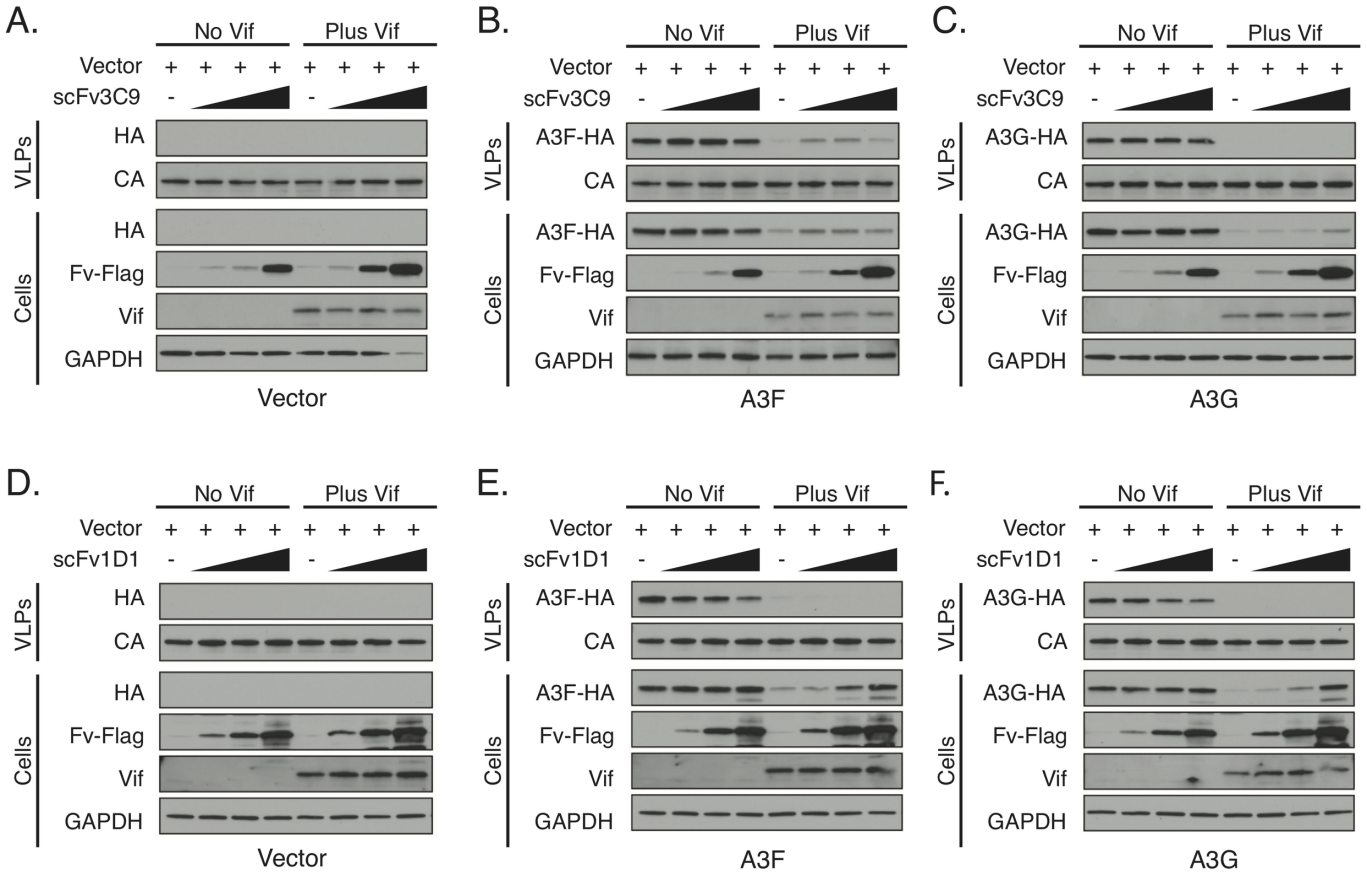
3C9 and 1D1 disrupt Vif-mediated A3 ubiquitination in cells but have differential effects on packaging into virus-like particles (VLPs). Immunoblot analysis of cellular A3 levels and packaging into VLPs in presence of Vif and Fab derived scFvs. Env-deficient NL4-3 HIV (either Vif+ or Vif-) was co-transfected into HEK293T cells with HA-tagged A3 and a gradient of Flag-tagged scFv3C9 (A-C) or scFv1D1 (D-F). The A3 proteins, Fabs, and Vif were visualized by WB using anti-HA, anti-Flag, and anti-Vif. p24 and GAPDH were used as loading controls for the VLP and cell fractions, respectively.

Having observed the ability of 3C9 and 1D1 to inhibit A3 ubiquitination *in vitro*, we next asked whether these Fabs could inhibit Vif-mediated degradation of A3s in cells. Accordingly, single-chain variable fragment constructs (scFv3C9 and scFv1D1) were generated for intra-cellular expression and tested for their ability to disrupt Vif-mediated degradation of A3C, A3F, and A3G (Fig 3, S3 and S4 Figs). Consistent with the *in vitro* ubiquitination data, scFv3C9 had no effect on Vif-mediated degradation of A3G, but was able to partially restore the levels of A3F (Fig 3A–3C). The restored A3F was packaged into viral like particles (VLPs), a critical requirement for A3-mediated restriction of HIV infection (Fig 3B). Notably, we do not see full restoration of A3F to levels observed in the absence of Vif nor do we find this amount of restored A3F to be sufficient to inhibit HIV replication in single cycle replication assays. This is potentially because we use the smaller scFv with full-length A3F in our cellular assays as opposed to the full-length Fab with the smaller C-terminal domain of A3F in our *in vitro* ubiquitination assay. In addition to A3F, we also investigated Vif-mediated degradation of A3C, as A3F and A3C bind a common surface of Vif [25,26]. Similar to A3F, scFv3C9 restored A3C cellular levels, further supporting the inhibitory role of 3C9 within cells (S4 Fig). Unlike A3F, A3C is not packaged into VLPs; therefore, despite scFv3C9 restoring A3C cellular levels, we do not expect the restored A3C to be packaged into the VLPs.

scFv1D1 was able to fully restore cellular levels of A3C, A3F, and A3G, demonstrating that 1D1 is able to block Vif-mediated ubiquitination of all tested A3s (Fig 3D–3F, S4 Fig). Surprisingly, the restored A3s were not packaged into VLPs, suggesting that the mechanism of inhibition for 1D1 is not therapeutically viable and that Vif has a degradation-independent mechanism for restricting A3 proteins. Together these data suggest that VCBC-targeted therapeutics should be screened for their ability to restore A3 packaging rather than for their ability to inhibit A3 ubiquitination. Moreover, these results support the development of inhibitors that can selectively target independent A3 binding sites.

### 3C9 is a non-competitive inhibitor of A3F ubiquitination

**Fig 4:**
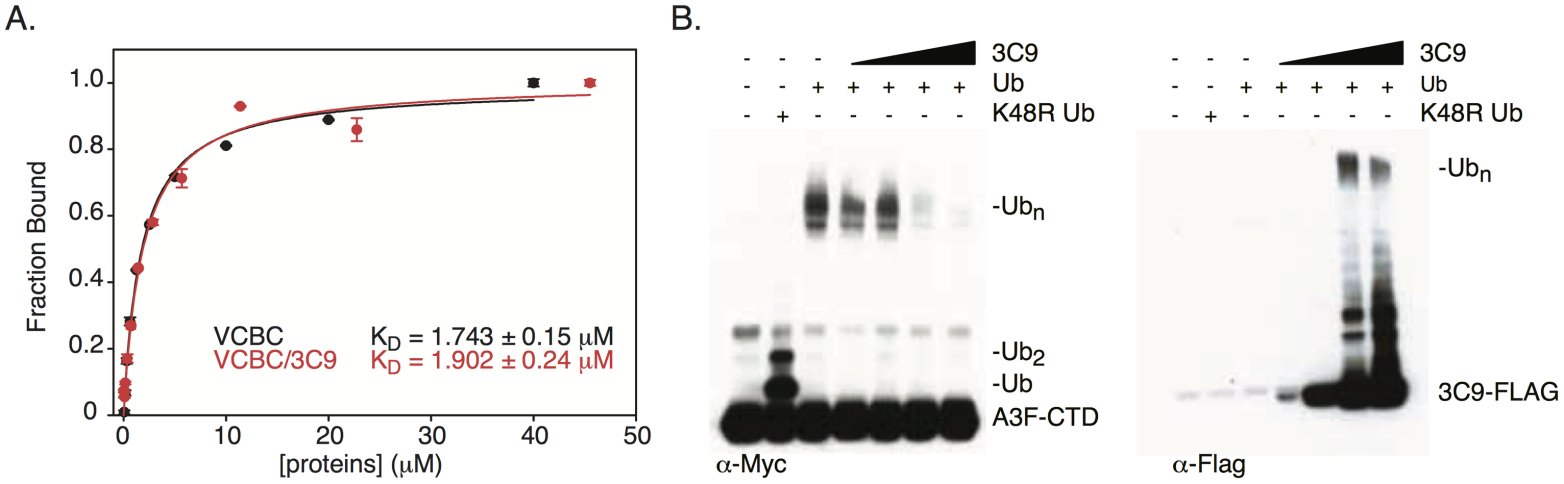
3C9 is a non-competitive inhibitor of Vif-mediated A3F-CTD ubiquitination. (A) Fluorescence polarization binding assay for VCBC binding to fluorescently labeled A3F-CTD in the absence (black) or presence (red) of 3C9. (B) *In vitro* ubiquitination of Myc-tagged A3F-CTD in the presence of increasing amounts of Flag-tagged 3C9. A3F-CTD and 3C9 were visualized by WB using anti-Myc and anti-Flag, respectively.

3C9 selectively inhibits A3F ubiquitination and partially rescues packaging into VLPs. The simplest mechanism to explain these effects is a competition between 3C9 and A3F for VCBC. To test this possibility, we performed several experiments, which instead showed that 3C9 inhibits A3F ubiquitination in a non-competitive manner. First, we performed direct binding and competition fluorescence polarization (FP) assays and found that the presence or absence of 3C9 had no affect on the VCBC/A3F-CTD interaction. For the direct binding assay, fluorescently labeled A3F-CTD was titrated with either VCBC or VCBC-3C9, and the K_D_ values for the interactions were found to be nearly identical (Fig 4A). For the competition assay, we pre-bound labeled A3F-CTD with VCBC and titrated this complex with either 3C9 or unlabeled A3F-CTD. 3C9 could not compete with labeled A3F-CTD for VCBC binding in contrast to the unlabeled A3F-CTD control (S5A and S5B Fig). Second, 3C9 and A3F-CTD were able to simultaneously bind VCBC and form a stable complex by SEC (S5C Fig).

Cullin-RING E3 ligases catalyze ubiquitin transfer by orienting substrate lysines toward an activated E2~Ub thioester that is bound by the RING-Box subunit [27,28]. One possible explanation for how 3C9 prevents A3F ubiquitination is that 3C9 sterically blocks ubiquitin transfer to A3F. We tested this possibility by assessing Vif-mediated ubiquitination of Myc-tagged A3F-CTD in the presence of Flag-tagged 3C9. This assay indicated that A3F-CTD and 3C9 have reciprocal poly-ubiquitination patterns: when A3F-CTD poly-ubiquitination was lost, 3C9 poly-ubiquitination appeared (Fig 4B). We conclude that 3C9 inhibits Vif-mediated ubiquitination of A3F by “shielding” it from ubiquitin transfer, and not by disrupting the VCBC-A3F interaction or precluding the formation of the Vif E3-ligase.

### 1D1 competes with CUL5 for VCBC binding

**Fig 5:**
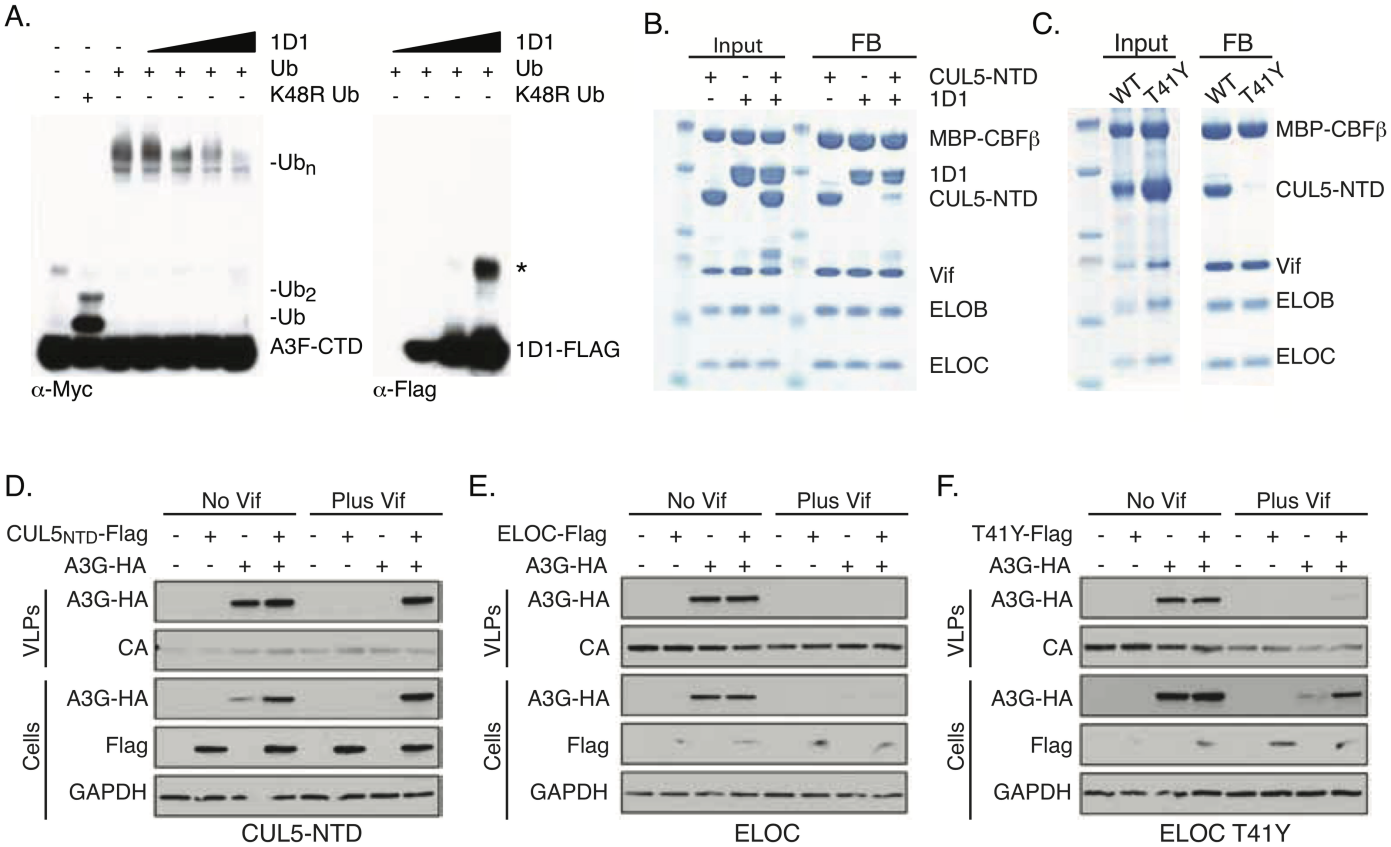
1D1 disrupts VCBC-CUL5 interaction to prevent A3 packaging. (A) *In vitro* ubiquitination of Myc-tagged A3F-CTD in the presence of increasing amounts of Flag-tagged 1D1. A3F-CTD and 1D1 were visualized by WB using anti-Myc and anti-Flag, respectively. The asterisk indicates non-reduced 1D1. (B) MBP-tagged VCBC was immobilized on amylose resin and incubated with 1D1 and/or CUL5-NTD. (C) MBP-tagged VCBC WT or VCBC containing ELOC T41Y were immobilized on amylose resin and incubated with CUL5-NTD. Input and final bead (FB) samples were resolved by SDS-PAGE gel and show proteins present before and after wash steps. (D-F) Env-deficient NL4-3 HIV (either Vif+ or Vif-) was co-transfected with HA-tagged A3G and Flag-tagged (D) CUL5-NTD, (E) ELOC WT (F) ELOC T41Y. A3G levels were measured in the cell and VLPs by WB with anti-HA.

Taking a similar approach as we did with 3C9, we assessed the Vif-mediated ubiquitination of Myc-tagged A3F-CTD in the presence of Flag-tagged 1D1. In contrast to 3C9, 1D1 was not ubiquitinated even though it inhibited A3F-CTD ubiquitination (Fig 5A). 1D1 may disrupt formation of the Vif E3-ligase since it is able to inhibit the ubiquitination of multiple A3s and is not ubiquitinated itself. Accordingly, we tested the ability of 1D1 to directly compete with CUL5 for VCBC binding. Pulldown experiments established that VCBC was able to interact with 1D1 and CUL5-NTD individually; however, when both 1D1 and CUL5-NTD are present, 1D1 prevented CUL5-NTD from binding to VCBC (Fig 5B).

Multiple groups have shown that disrupting A3 ubiquitination by mutating the A3 binding surface on Vif, by expressing a dominant negative CUL5 variant, or by pharmacologically inhibiting the E3-ligase restores cellular A3 levels and results in A3 packaging [3,15,29]. Similarly, 3C9 prevents A3F degradation and allows for rescued A3F packaging. 1D1, on the other hand, restores the cellular levels of A3, but the rescued A3 is unable to be packaged. We hypothesized that 1D1’s ability to prevent A3 packaging was associated with its ability to disrupt the VCBC-CUL5 interaction. To test this, we assessed the levels of A3G in VLPs in the presence of CUL5-NTD, ELOC, or ELOC T41Y mutant which disrupts VCBC-CUL5 binding (Fig 5C–5F) [30]. CUL5-NTD restored the levels of A3G within the cell and VLPs, indicating that the N-terminal domain of CUL5 was sufficient to block A3 ubiqutination and restore A3 packaging (Fig 5D). In contrast, ELOC T41Y only restored cellular A3G levels and, like 1D1, prevented A3G packaging. This can be attributed to the loss of CUL5 binding, since overexpression of WT ELOC has no effect of A3G levels (Fig 5E and 5F). Collectively, these data support a model in which Vif uses a degradation-independent mechanism to prevent A3 packaging in the absence of CUL5 binding.

### Structure of the VCBC-3C9-1D1 complex

**Fig 6:**
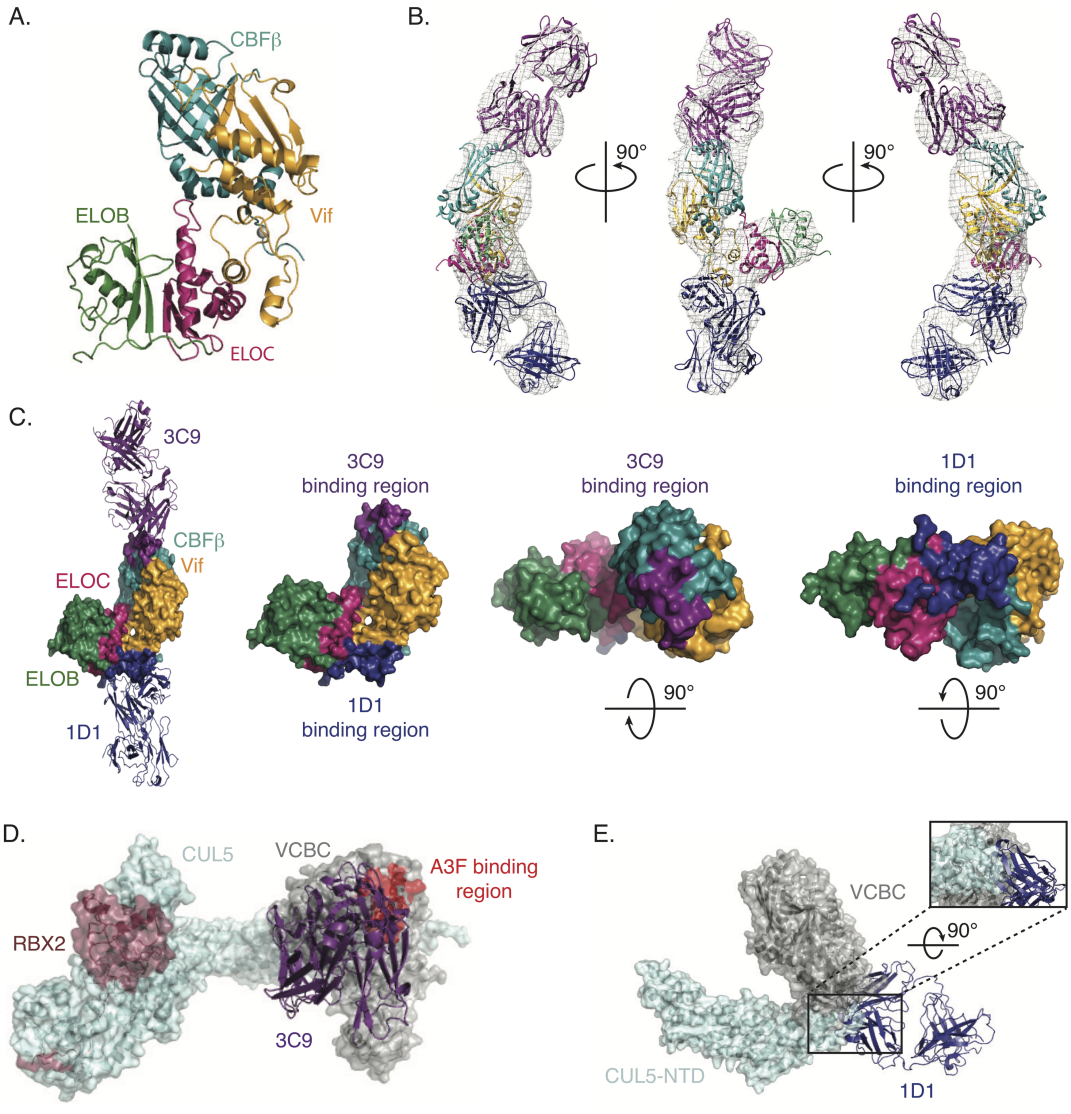
EM model of VCBC/3C9/1D1. (A) Ribbon representation of the VCBC complex from the VCBC-CUL5-NTD crystal structure (PDB: 4N9F). (B) VCBC and homology models of 3C9 and 1D1 fit into a representative EM map. (C) 3C9 and 1D1 binding surfaces on VCBC are shown in purple and blue, respectively. (D) A3F binding region (red) highlighted onto the 3C9/Vif E3-ligase model. Vif E3-ligase model was generated by aligning a full-length CUL5/RBX2 model on the VCBC-CUL5-NTD structure. (E) Alignment of the VCBC-CUL5-NTD complex (PDB: 4N9F) and the VCBC-1D1 complex from the VCBC-3C9-1D1 NSEM model. Inset shows the predicted steric clashes between 1D1 and CUL5.

To determine the structural basis for VCBC antagonism by 3C9 and 1D1, we used negative stain electron microscopy (NSEM) to define the VCBC-3C9-1D1 complex at low resolution (Fig 6). Initial micrographs and Two dimensional (2D) class-averages revealed a homogeneous sample with well-defined particles that enabled the reconstruction of a 3D structure (S6 Fig). 3D classification yielded two EM maps with very similar overall structural features. The densities with the characteristic Fab shape provide a validation to these two EM maps. Structural alignments revealed that the maps differ due to flexibility within VCBC that is likely caused by a “hinge” region connecting the CBFβ-binding domain of Vif to the ELOC-binding “BC box” (S7A–S7C Fig). Of note, the “hinge” region of Vif is comprised of three loops and a zinc-finger motif, which coordinates Zn^2+^ to help stabilize this inter-domain region of Vif.

In constructing a molecular model, the crystal structure of VCBC (PDB: 4N9F) and homology models of 3C9 and 1D1 were fitted into the EM map (Fig 6A and 6B). The specific assignment of 3C9 and 1D1 into the fab shaped densities could not be immediately discerned; however, multiple lines of evidence guided us to the correct placement of each Fab. First, 2D class averages of VCBC-3C9-3F12, VCBC-1D1-3F12, VCBC-3C9-3F12, and VCBC-3C9-3F12-1D1 complexes reveal that 3F12 and 1D1 bind to opposite sides of VCBC than 3C9 (S8A Fig). Second, we have shown that 3F12 binds an epitope within ELOB, and therefore, 3C9 binds opposite of 3F12 near the Vif/CBFβ interface and 1D1 binds near ELOC (S8 Fig). Finally, biochemical and cell based studies indicate 1D1 competes with CUL5 for binding VCBC, consistent with it binding near ELOC (Fig 5B–5F).

The NSEM data revealed the 3C9 and 1D interacting patches on the surface of VCBC, and both Fabs appear to bind VCBC across composite protein-protein interfaces (Fig 6C). The 3C9 epitope is located along the Vif/CBFβ interface, such that CBFβ would contribute the major interacting surface, while the 1D1 epitope is positioned evenly across the Vif/ELOC interface. To evaluate if 3C9 and 1D1 recognize epitopes on Vif, host factors or combinations of both, we performed SEC to determine whether 3C9 and 1D1 can recognize VCBC components in the absence of Vif. In this assay, we assessed the ability of 3C9 and 1D1 to bind CBFβ alone or ELOBC in complex with its host-binding partner SOCS4. Though 3C9 and 1D1 can bind to VCBC, neither Fab bound to CBFβ or ELOBC-SOCS4, indicating that Vif is required for Fab interaction (S9 Fig).

To identify structural regions that may contribute to 3C9 “shielding” we modeled full-length CUL5/RBX2 on the VCBC-3C9-1D1 structure and highlighted Vif residues known to be important to A3F binding (Fig 6D). Indeed, this structural analysis suggests that 3C9 can sterically “shield” A3F-CTD from the UB-E2, which is in agreement with our current biochemical and enzymologic data. To determine the structural basis for 1D1 inhibition, we aligned the VCBC-3C9-1D1 structure with the previously reported VCBC-CUL5-NTD structure (Fig 6E). Analysis of these two structures predicts a steric clash between 1D1 and CUL5, and explains why 1D1 is able to compete with CUL5 for VCBC binding.

## Discussion

Several studies have indicated that inhibition the Vif E3-ligase complex can restore A3 restriction and block viral replication, but there are no known drugs to date that directly target Vif. In this study, we developed Fabs that bind to the HIV-1 VCBC complex and demonstrated the utility of these Fabs as selective reagents to probe Vif function. Our panning campaign resulted in six Fabs that bound to VCBC with high affinity, of which 3C9, 3F12, and 1D1 were selected for additional studies as they recognize unique epitopes on VCBC. We initially categorized the Fabs based on their ability to disrupt Vif-mediated A3 ubiquitination, as Fabs that disrupt A3 ubiquitination would provide valuable information regarding Vif inhibition strategies, and Fabs that do not perturb A3 ubiquitination may be useful reagents for structural studies. Indeed, we have isolated Fabs that have no effect on A3 ubiquitination (3F12), inhibit A3F/A3C ubiquitination (3C9), or inhibit all A3 ubiquitination (1D1).

We show that 3C9 is able to selectively inhibit the ubiquitination of A3F/A3C, but not A3G, indicating the feasibility of targeting specific A3 sites on Vif (Fig 3). Importantly, the restored cellular A3F was packaged into VLPs, suggesting that 3C9 may provide valuable insight into the development of Vif inhibitors. Through a combination of structural and biochemical studies, we discovered that 3C9 inhibits A3F ubiquitination in a non-competitive manner, and we proposed a model where 3C9 shields A3F from ubiquitin transfer without perturbing assembly of the Vif E3-ligase complex. This is supported by the fact that the Vif E3-ligase is catalytically competent to ubiquitinate 3C9 in place of A3F. The shielding model may also explain why the smaller scFv3C9 only partially restored full-length A3F in the cell-based assay.

We identified a second biologically relevant Fab, 1D1, which acts as a pan-inhibitor of A3 ubiquitination. Structurally and biochemically, we have shown that 1D1 disrupts the VCBC-CUL5 interaction, thereby preventing A3 ubiquitination and degradation. Initially, this seemed like a promising strategy to antagonize Vif as it would 1) restore the levels of all A3 proteins degraded by Vif, 2) be potentially amenable to small molecule inhibition as the Vif-CUL5 interface is the smallest protein-protein interface in the Vif complex, and 3) may avoid off-target effects as Vif uses a non-canonical Cullin-binding motif to interact with CUL5 [30–33]. To our surprise, however, 1D1 did not rescue A3 packaging, which is a critical step for A3 to restrict HIV infection. Therefore, targeting the Vif-CUL5 interface is not a viable strategy for restoring the restrictive properties of A3s, and high-throughput screens that monitor cellular A3 levels may not provide therapeutically viable Vif inhibitors.

**Fig 7:**
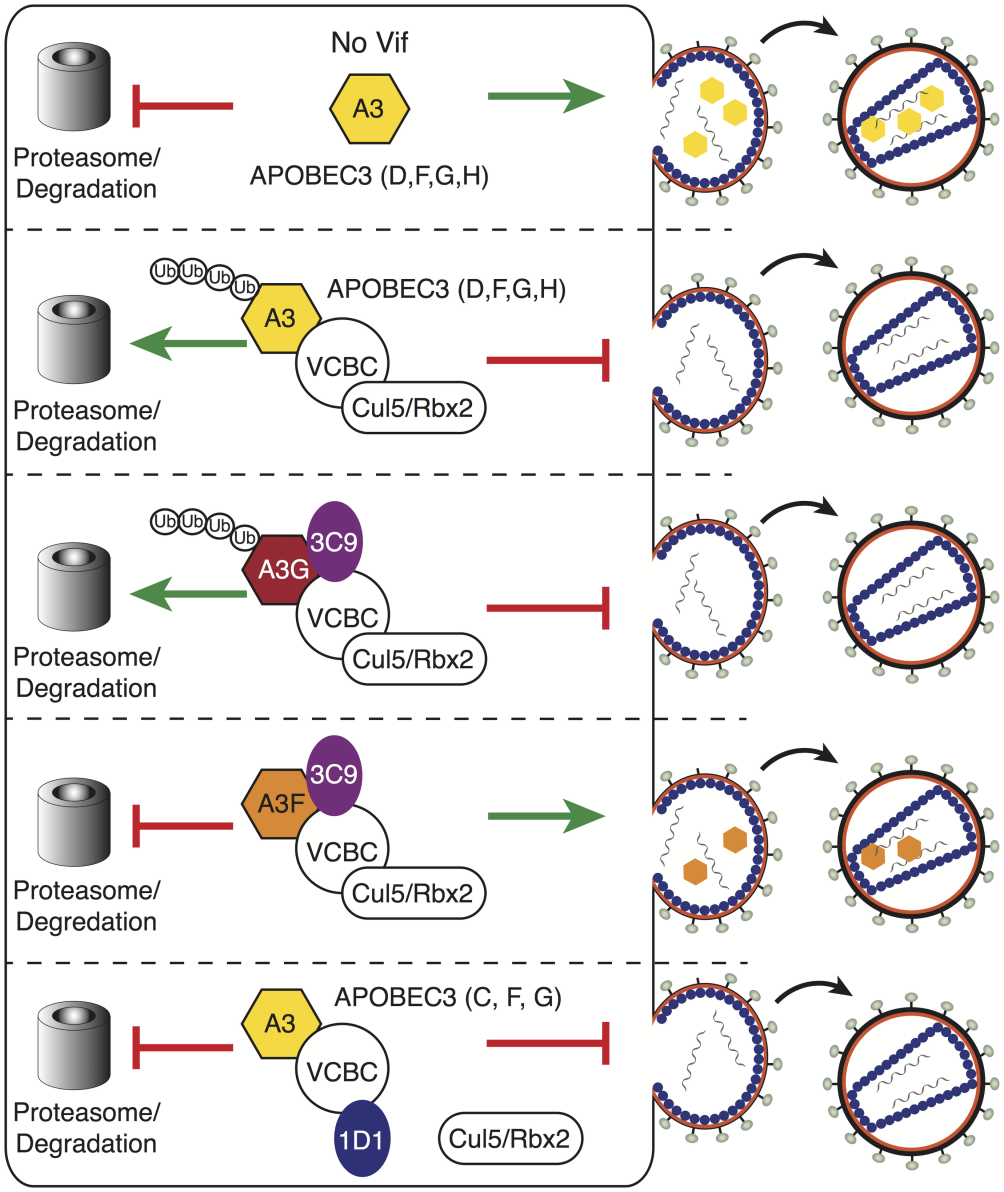
Fab mechanisms of inhibition. In the absence of Fabs, the Vif E3 ligase is able to ubiquitinate A3 proteins (A3D, F, G, H) and target them for proteasomal degradation. 3C9 inhibits the ubiquitination and degradation of A3F, but not A3G, and rescues A3F packaging. 1D1 prevents the ubiquitination and degradation of A3, and inhibits A3 packaging.

The prevalent mechanism for Vif-mediated antagonism of A3 is through ubiquitination and proteasomal degradation. Previous studies investigating A3 mutants and cell-to-cell variability have suggested that a degradation-independent mechanism exists [11,34–37]. Despite these studies, a degradation-independent mechanism for Vif has gained little recognition over the last 15 years because it has been hard to demonstrate conclusively: CUL5 knock-downs are toxic and CUL5 dominant-negative constructs are sufficient to restore A3 packaging. Here we use 1D1, as well as an ELOC mutant, to show that Vif has a degradation-independent mechanism to antagonize A3 in the absence of CUL5 binding (Fig 7). It is currently unclear why the presence of CUL5 releases A3 from sequestration by VCBC. In principle, CUL5 could directly facilitate A3 packaging by binding to VCBC and inducing substrate release; or alternatively, CUL5 could act indirectly by bringing additional binding partners to the VCBC/A3G complex that induce A3 release. Though additional studies will be needed to elucidate the role of CUL5 in substrate release, our data establishes that Vif can prevent A3 packaging in a degradation-independent manner.

In summary, we have developed a diverse panel of Fabs for use as biochemical probes to better understand Vif antagonism of the innate immunity provided by A3 restriction factors. These studies support the feasibility of pursuing Vif as a therapeutic target, but indicate that inhibition of ubiquitination by the Vif E3-ligase alone may not be sufficient to rescue A3 packaging. Importantly, our data establish an degradation-independent mechanism by which Vif prevents A3 packaging, and this together with previous studies show that Vif repression of A3 packaging is a multilayered process involving both degradation dependent and independent mechanisms [9,22,29,38,39]. The observations we have made here to further support Vif’s multiple approaches to antagonize A3 are consistent with the fact that HIV and many other viruses, especially those with a limited number of genes, encode for multifunctional proteins. Given the number of viral proteins that hijack host ubiquitin machinery to carry out their functions, it is plausible that other viral proteins may use multi-layered approaches, similar to Vif, to efficiently suppress innate immune responses.

## Materials and methods

### Protein expression and purification

All constructs were generated by standard PCR and restriction-based cloning methods unless otherwise noted. For mammalian expression, scFv3C9 and scFv1D1 with C-terminal 3x-Flag tags were cloned into pcDNA4 (Invitrogen). Human A3C, A3F, and A3G expression constructs with C-terminal 3xHA tags have been previously reported [40]. Env-deficient HIV-1 NL4-3 full molecular clones were kindly provided by the lab of Alan Frankel (UCSF). ELOC was cloned into pcDNA4/TO (Invitrogen) with an N-terminal 3xFlag tag and site-directed mutagenesis was used to engineer the T41Y mutant. Likewise, the N-terminal domain of CUL5 (amino acids 1–386) containing two mutations (V341R and L345R) was cloned into pcDNA4/TO (Invitrogen) with a C-terminal 3xFlag tag.

All proteins, except A3G, were expressed in *E*. *coli*. Recombinant Fabs were expressed from phagemid as described [24]. HIS_6_-thioredoxin (Trx)-tagged CBFβ/Vif and ELOC/ELOB were co-expressed from pET-Duet and pCDF-Duet plasmids, respectively. HIS_6_-GB1-tagged CUL5-RBX2 was co-expressed from a pRSF-Duet plasmid. SOCS4 and ELOC/ELOB were co-expressed from pET28a and pCDF-Duet plasmids, respectively. HIS_6_-Trx-myc-tagged A3F-CTD was expressed from a pHisTRX plasmid. For *E*. *coli* expression, plasmids were transformed into *E*. *coli* BL21(DE3) (Invitrogen) cells and grown at 37°C to an optical density of 0.6–0.8 and induced with 0.5 mM IPTG overnight at 18°C (or 1mM IPTG at 20°C for Fabs).

Purified VCBC, 6-protein CRL5^Vif-CBFβ^ complex, and the SOCS4-ELOBC complex were obtained as described previously [9,22]. Fabs were purified from the periplasmic fraction following protocols described previously [41]. Briefly, all proteins were subjected to Ni-NTA affinity purification followed by size exclusion chromatography as a final purification step. All tags were removed by TEV protease prior to SEC. Vif complexes were stored in 20mM HEPES pH 8, 300 mM NaCl, 10% glycerol, and 1mM DTT. Fabs were stored in 1x PBS buffer containing 10% glycerol. To obtain VCBC-Fab complexes, VCBC and Fabs were incubated on ice for 10min and then purified by SEC.

A3G was C-terminally myc-tagged followed by a TEV cleavage site and GFPII and expressed in baculovirus infected Sf9 cells. Purification was carried out as previously described [42]. Minor modifications include use of Straptactin affinity resin and TEV protease to remove the GFP tag prior to size-exclusion chromatography. A3G was stored in 20mM Tris (pH7.5), 500mM NaCl, 10% glycerol, and 1mM DTT.

### Phage display panning

We used a previously published human naïve B cell phage library with a diversity of 4.1 x 10^10^ to identify Fabs against VCBC (23). Fabs were isolated using a phage display panning protocol descirbed in detail by Kim et al, 2011 [24]. Briefly, purified VCBC was biotinylated using EZ-Link NHS-Chromogenic-Biotin (Pierce) as described previously [24]. A fully human naïve Fab phage display library was used to identify antibodies against VCBC complex [23,24]. The panning was accomplished in four rounds using streptavidin magnetic beads (Invitrogen) coated with biotinylated VCBC complex as described previously. After four rounds of selection, Fabs that bind to VCBC were identified by an ELISA screen [41]. Clones with a positive signal in ELISA were analyzed by BstNI restriction analysis to identify the unique clones. scFvs were generated by linking the heavy and light chain variable regions together with a 24 aminoacids linker (ASSGGSTSGSGKPGSGEGSSGSAR), and then cloning these constructs into pSYN1 or pcDNA4, for E.coli or mamalian expression, respectively.

### Qualitative and competitive ELISA

The ELISA experiments were done as described previously [24]. All the Fabs expressed from our phage display library have a myc tag in the C-terminus of the heavy chain that is used for the detection of the Fab in the ELISAs. For Competitive ELISAs, we need two different tags for independent detection of the two competing Fabs. The Myc tag in the phagemid was replaced with the Flag tag using standard cloning methods [41].

### BioLayer Interferometry (BLI)

Kinetic constants for VCBC Fabs were determined using an Octet RED384 instrument (ForteBio). Four concentrations of each Fab were tested for binding to the biotinylated VCBC complex immobilized on ForteBio streptavidin SA biosensors. All measurements were performed in 20 mM HEPES pH 7.4, 0.5 M NaCl with 0.1% (w/v) bovine serum albumin (BSA) and 0.02% (v/v) Tween 20 at room temperature in 384-well microplates. Data were analyzed using a 1:1 interaction model on the ForteBio data analysis software 8.2.

### *In vitro* ubiquitination assay

Vif-mediated *in vitro* ubiqutination of A3F-CTD and A3G were done as previously reported [9]. Briefly, the ubiquitination assays were performed for 1 hour at 25°C with 300 nM NEDD8ylated HIV-1_NL4-3_ E3 holoenzyme, 2–4 μM myc-tagged A3F-CTD or myc-tagged A3G, and the ubiquitin activating system containing: 1.2 μM ATP, 1.2 μM ubiquitin activating enzyme (UBE1), 45 μM ubiquitin, 4.8 μM E2 (CDC34). Ubiquitination reactions done in the presence of Fabs contained 0.1 μM, 0.5 μM, or 1μM of indicated Fab. Ubiquitinated A3 proteins were detected using a monoclonal anti-c-myc antibody (Sigma).

### Cell lines

Human Embryonic Kidney (HEK) 293T cells (ATCC) were maintained in Dulbecco’s Modified Eagle Medium (DMEM) containing 10% fetal bovine serum (FBS) and 0.5% penicillin/streptomycin (P/S).

### A3 ubiquitination and packaging assay

At 50% confluency, HEK293T cells were transfected (TransIt, Mirus) with 1 μg Env-deficient HIV-1 NL4-3 proviral construct in the presence or absence of 25 ng of the indicated C-terminal HA-tagged APOBEC3 expression construct, and either 0, 50, 100, or 250 ng of the Flag-tagged scFv expression construct. Alternately, cells were transfected with 1 μg Env-deficient HIV-1 NL4-3 proviral construct in the presence or absence of 50 ng of the C-terminal HA-tagged APOBEC3G expression construct and in the presence or absence of 100 ng of the Flag-tagged CUL5 or ELOC expression construct. After 48 hours, viral supernatants and the cells were processed for immunoblotting. Cell lysates were prepared by resuspension of washed cell pellets directly in 2.5x Laemmli Sample Buffer (32.5mM Tris pH 6.8, 10% glycerol, 1.0% SDS, 2.5% 2-mercaptoethanol, 0.05% bromophenol blue), and homogenization at 95°C for 30 minutes. Virus-like particles were isolated from culture supernatants by purification through 0.45μm PVDF filters (Millipore) followed by centrifugation (13,000 rpm for 2 hours) through a 20% sucrose, 1x PBS cushion and lysis directly in 2.5x Laemmli Sample Buffer.

### Immunoblotting

Samples were run on 4–20% Tris-HCl SDS-PAGE gels (BioRad Criterion) at 150V for 90 minutes. Proteins were transferred to PVDF membranes by methanol-based electrotransfer (BioRad Criterion Blotter) at 90V for 2 hours. Membranes were blocked in 4% Milk in PBS, 0.1% Tween-20 overnight prior to overnight incubation with primary antibody against HA (HA.11; Covance), Vif (NIH ARRRP 809), Flag (Sigma F7425), GAPDH (Sigma G8795), p24/capsid (NIH ARRRP 3537 courtesy of B. Chesebro and K. Wehrly). Anti-mouse and anti-rabbit horseradish peroxidase (HRP)-conjugated secondary antibodies (BioRad) were detected using Hyglo HRP detection reagents (Denville Scientific). Blots were incubated in a 1xPBS, 0.2M glycine, 1.0% SDS, 1.0% Tween-20, pH 2.2 stripping buffer before reprobing.

### Fluorescence polarization assay

A3F-CTD was labeled with maleimide–conjugated fluorescein at 4°C for 12 hrs in 20 mM HEPES pH 7, 300 mM NaCl, 1 mM TCEP. For the direct binding assays, labeled A3F-CTD was diluted to 20nM and incubated with increasing amounts of VCBC or VCBC/3C9 for 20min at 25°C in 20 mM HEPES pH 8, 300 mM NaCl, 5% glycerol, 1 mM DTT. For the competition assays, VCBC was pre-bound with labeled A3F-CTD, and unlabeled A3F-CTD or 3C9 were titrated into the pre-assembled complex. Fluorescence polarization measurements were made using an Analyst plate reader (LJL Biosystems) with excitation and emission wavelengths of 485 nm and 530 nm, respectively. Titration assays were performed in duplicate, and all data were analyzed in SigmaPlot using equations derived as previously described [43].

### Negative stain electron microscopy

1D13C9-VCBC was diluted to a final concentration of 50 × 10^−9^ M. For all experiments, 3 μL of the sample was applied to a glow-discharged, carbon-coated, 400 mesh Cu grid and stained with 2% (wt/vol) uranyl formate as previously described [44]. Images were either acquired manually or using Leginon [45] at a nominal defocus range of 0.6–1.9 μm on a Tecnai 12 electron microscope (FEI, Eindhoven, Netherlands) operating at 120 kV. Images were recorded on a 4096 x 4096 CCD camera (Ultrascan 4000, Gatan, Pleasanton, CA) with a nominal magnification of either 52k or 67k corresponding to a pixel size of either 2.21 Å or 1.6 Å. For obtaining initial 2D class averages of VCBC fab complexes, particles were picked from 20–30 images. For 3D reconstruction particles were picked from 829 images.

### Image processing and reconstruction

Data was processed in one of two ways. For initial 2D class averages of VCBC fab combinations images were binned by a factor of two for particle picking and subsequent image processing. Individual particles were manually picked, extracted at a box size of 64 pixels and normalized to have a mean of 0 and a standard deviation of 1. Particles were subject to two-dimensional classification using Relion [46]. For 3D reconstruction experimental data were processed using the Appion software package [47], which interfaces with the Leginon database infrastructure. The contrast transfer function for each micrograph was estimated using CTFFIND4 [48]. Initial particle picking was done on a subset of micrographs using the DoG picker software [49], which uses a reference-free difference of Gaussians approach. To generate templates, these particles were sorted, aligned, and clustered using reference-free Clustering 2D (CL2D) algorithm implemented in Xmipp2 [50]. A subset of the resulting 2D class averages were used as references to re-pick particles on all micrographs. This yielded a total of 132,994 particles that were extracted at a box size of 256 pixels (binned by a factor of 2). CryoSPARC was used to sort particles into 10 ab initio 3D maps [51]. The three maps that contained recognizable features, which comprised 69822 particles, were subjected to an additional round of 3D classification into five different classes. The two resulting maps with the most convincing and consistent features were selected (one comprised of 18,503 particles and the other comprised of 19,969).

### Fab models

The PIGS server for the automatic prediction of antibody structures was used to identify fab structures with the highest sequence homology to 3C9 and 1D1 and to generated a predicted structure for the variable regions of 3C9 and 1D1 [52]. To obtain the predicted constant region of each fab, the Phyre2 server was used to thread the 3C9 and 1D1 sequences onto PDB ID 3H42 or 2J6E, respectively [53]. Combining the Phyre2 threaded constant region with PIG servers predicted variable region produced the final fab models.

### Pulldown assays

Pulldown assays were performed in buffer containing 20 mM HEPES pH 8, 300 mM NaCl, and 10% glycerol at 25°C. MBP-tagged VCBC WT or mutant proteins were immobilized on amylose resin. Resin bound MBP-tagged VCBC was incubated with excess purified CUL5-NTD and/or 1D1 protein and subsequently washed. Pulldown samples were separated on a SDS−PAGE gel and stained with coomassie blue dye.

## Supporting information

S1 Fig Consensus Vif supports HIV-1 replication. (A) Sequence alignment of HIV-1 NL4-3, HXB2, and consensus Vif. (B-C) HIV-1 infectivity data comparing WT and consensus Vif in the absence or presence of (B) A3G and (C) A3F.
(TIF)

S2 Fig Fab CDR sequences. Primary sequences for the (A) light and (B) heavy chain CDRs for Fabs 1D1 and 3C9.
(TIF)

S3 Fig scFv3C9 and scFv1D1 inhibit A3 ubiquitination in vitro. (A) Cartoon depiction of an antibody, a fab, and a scFv. The heavy (blue) and light (orange) chain constant and variable regions are highlighted in the fab and scFv. (B-D) *In vitro* ubiquitination of Myc-tagged A3F-CTD or A3G in the absence or presence of increasing amounts of (B) 3C9 and scFv3C9 or (C-D) 1D1 and scFv1D1.
(TIF)

S4 Fig 3C9 and 1D1 disrupt Vif-mediated A3C ubiquitination in cells. Env-deficient NL4-3 HIV (either Vif+ or Vif-) was co-transfected with A3C and a gradient of (A) scFv3C9 or (B) scFv1D1.
(TIF)

S5 Fig 3C9 does not compete with A3F for VCBC interaction. (A) Fluorescence polarization competition assay. Pre-bound fluorescently labeled A3F-CTD with VCBC was titrated with either 3C9 (red) or unlabeled A3F-CTD (black). (B) Simulated competition curves establish that 10 μM of 3C9 and 100 μM of A3F-CTD are sufficient to compete with the labeled A3F-CTD bound to VCBC. (C) SEC elution profile for VCBC/3C9/Trx-A3F-CTD (black), VCBC/3C9 (red), VCBC/Trx-A3F-CTD (blue), VCBC (green), Trx-A3F-CTD (orange). SDS-PAGE gel of peak fraction for VCBC/3C9/Trx-A3F-CTD.
(TIF)

S6 Fig VCBC-3C9-1D1 NSEM sample. (A) SEC profile and corresponding SDS-PAGE gel for VCBC-3C9-1D1. (B) Representative NSEM micrograph and (C) 2D class averages for VCBC-3C9-1D1.
(TIF)

S7 Fig VCBC/3C9/1D1 NSEM 3D classification. EM maps aligned on (A) 1D1 or on (B) 3C9 reveal that the two conformations arise due to flexibility in VCBC. (C) The CBFβ and ELOC binding regions of Vif are boxed off. Postulated “hinge” connects these two regions and is comprised of the Zn+ binding motif and three interdomain loops.
(TIF)

S8 Fig NSEM 2D class averages and 3F12 epitope mapping determine Fab organization. (A) NSEM 2D class averages and corresponding cartoon depictions of VCBC-Fab complexes show that 3F12 and 1D1 bind the same side of VCBC, and that 3C9 binds the opposite side of VCBC from 1D1 and 3F12. (B) SD200 elution profile and corresponding SDS-PAGE gels show that 3F12 is able to form a stable complex with SOCS4/ELOBC. (C) Purified VCBC complex resolved by SDS-PAGE gel. Myc-tagged Fabs were used as a primary antibody to WB for individual VCBC components. 3F12 is able to detect ELOB in a WB. The asterisk indicates a non-specific protein.
(TIF)

S9 Fig Vif is required for co-purification of 3C9 and 1D1 with VCBC. (A) SD200 elution profile and corresponding SDS-PAGE gels for (B) SOCS4/ELOBC and SOCS4/ELOBC in the presence of (C) 3C9 or (D) 1D1. (E) SD75 elution profile and corresponding SDS-PAGE gels for (F) CBFβ and CBFβ in the presence of (G) 3C9 or (H) 1D1.
(TIF)
