# Supplementary figures and images for: Fab-based inhibitors reveal ubiquitin independent functions for HIV Vif neutralization of APOBEC3 restriction factors
Source: PLoS Pathog. 2018 Jan 5;14(1):e1006830. doi: 10.1371/journal.ppat.1006830 (PMC5773222; doi:10.1371/journal.ppat.1006830)

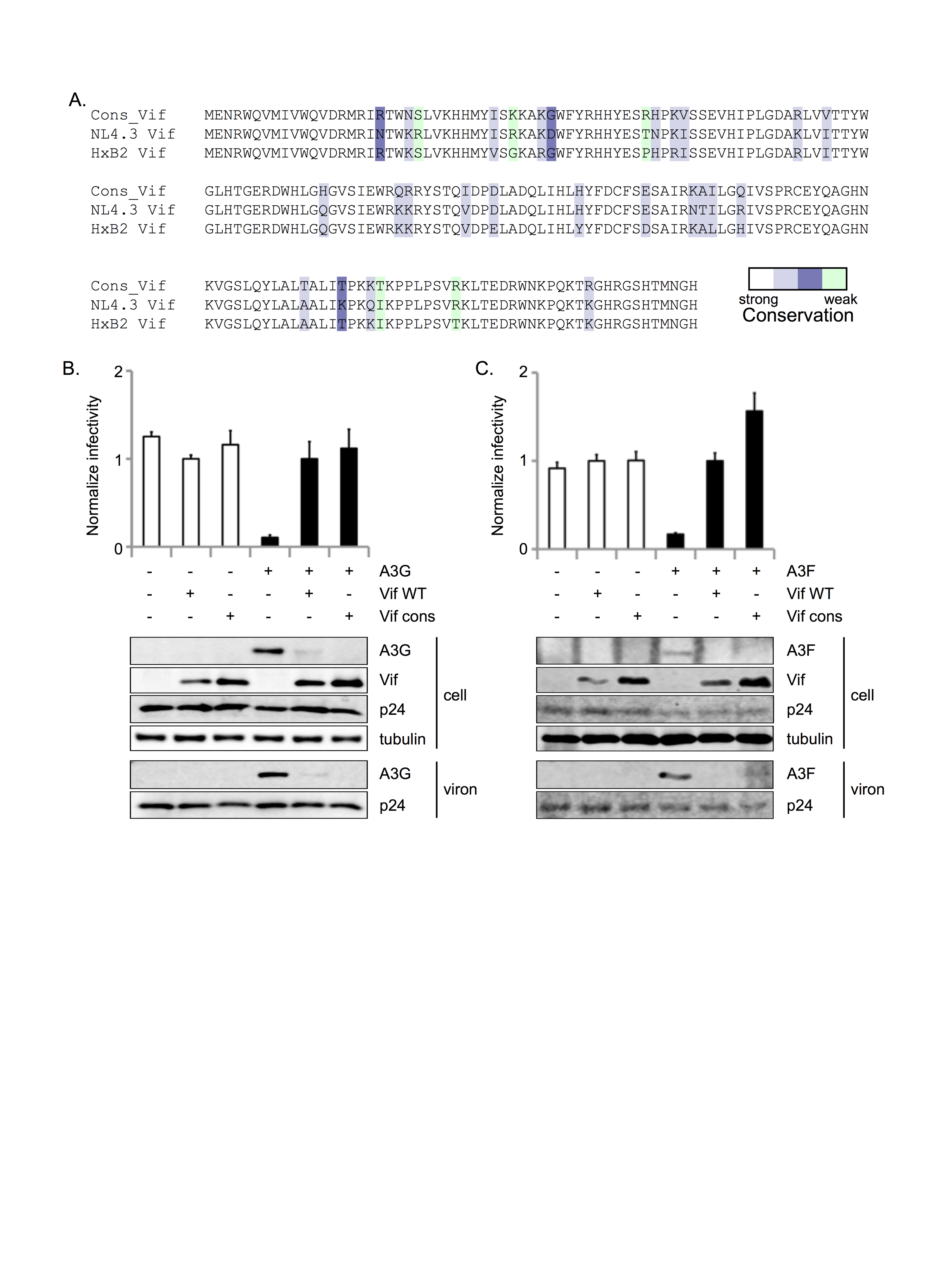

Supplement: S1 Fig — (A) Sequence alignment of HIV-1 NL4-3, HXB2, and consensus Vif. (B-C) HIV-1 infectivity data comparing WT and consensus Vif in the absence or presence of (B) A3G and (C) A3F. (TIF) [file ppat.1006830.s001.tif]

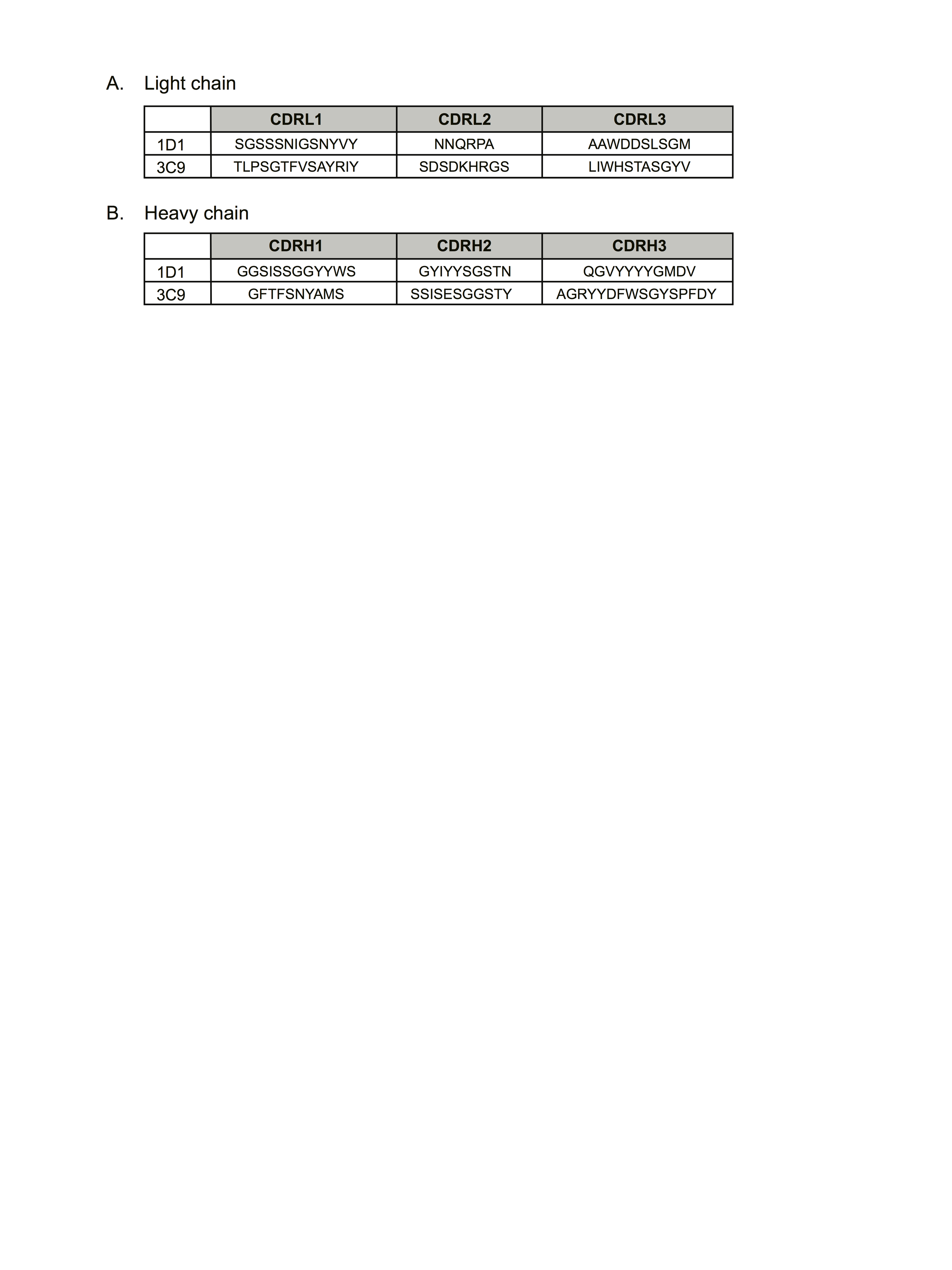

Supplement: S2 Fig — Primary sequences for the (A) light and (B) heavy chain CDRs for Fabs 1D1 and 3C9. (TIF) [file ppat.1006830.s002.tif]

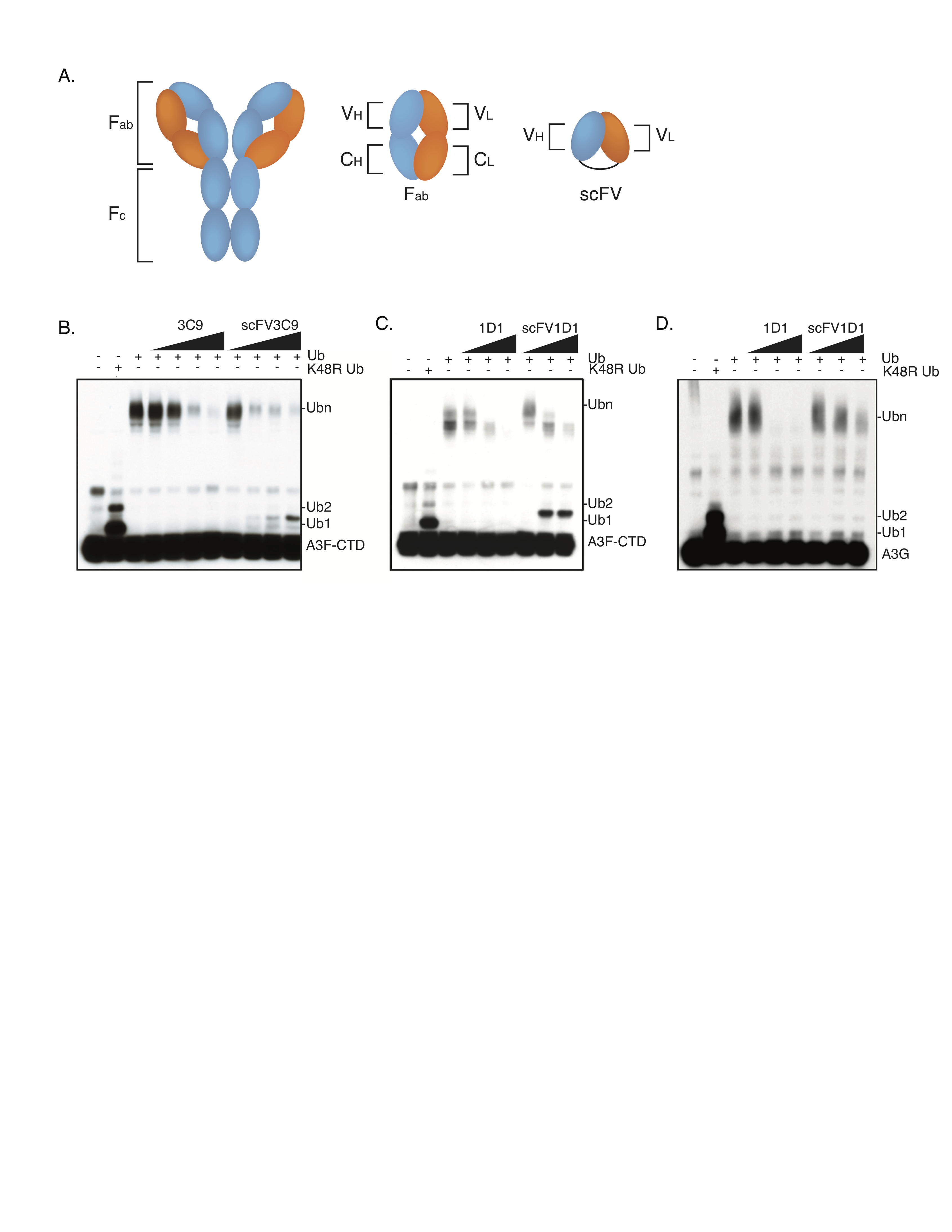

Supplement: S3 Fig — (A) Cartoon depiction of an antibody, a fab, and a scFv. The heavy (blue) and light (orange) chain constant and variable regions are highlighted in the fab and scFv. (B-D) In vitro ubiquitination of Myc-tagged A3F-CTD or A3G in the absence or presence of increasing amounts of (B) 3C9 and scFv3C9 or (C-D) 1D1 and scFv1D1. (TIF) [file ppat.1006830.s003.tif]

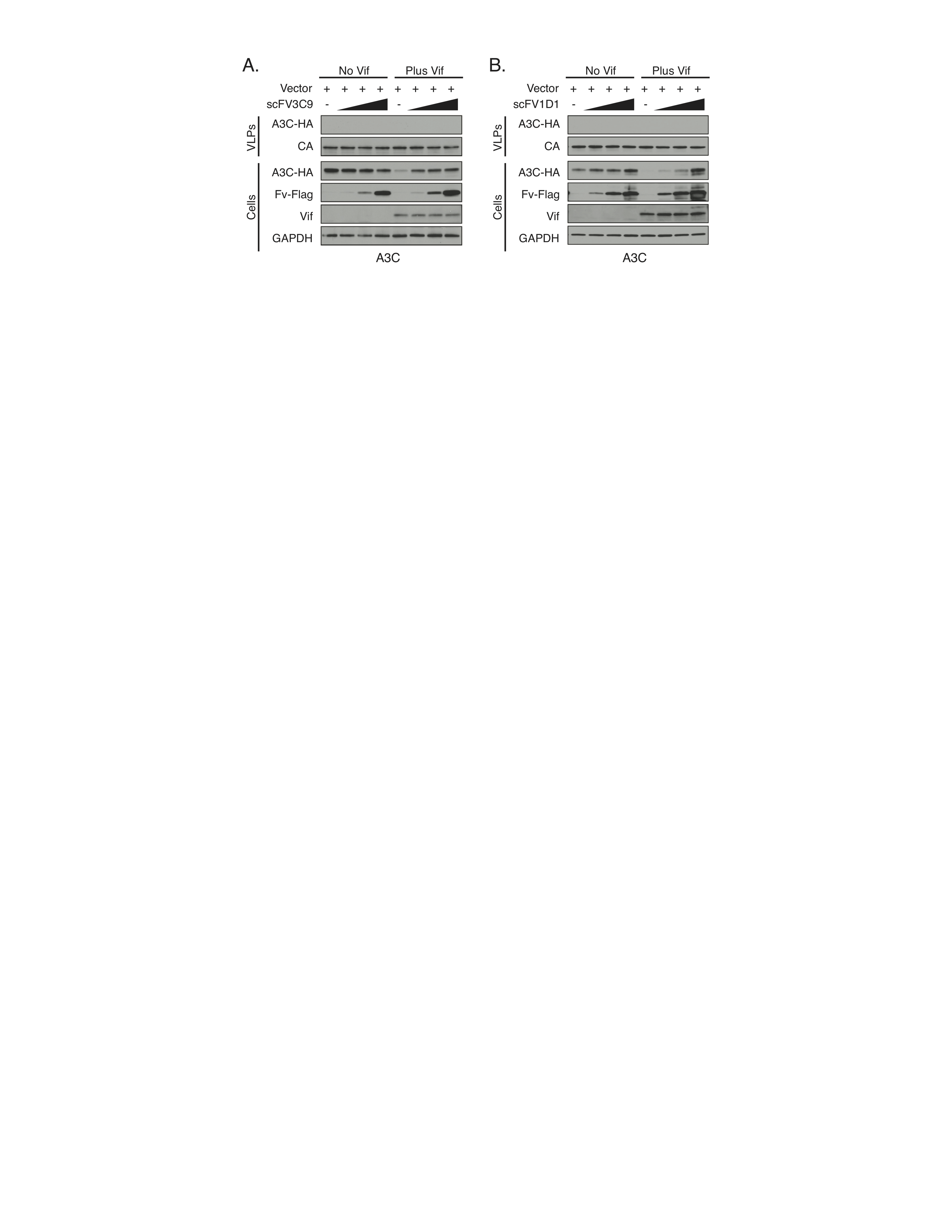

Supplement: S4 Fig — Env-deficient NL4-3 HIV (either Vif+ or Vif-) was co-transfected with A3C and a gradient of (A) scFv3C9 or (B) scFv1D1. (TIF) [file ppat.1006830.s004.tif]

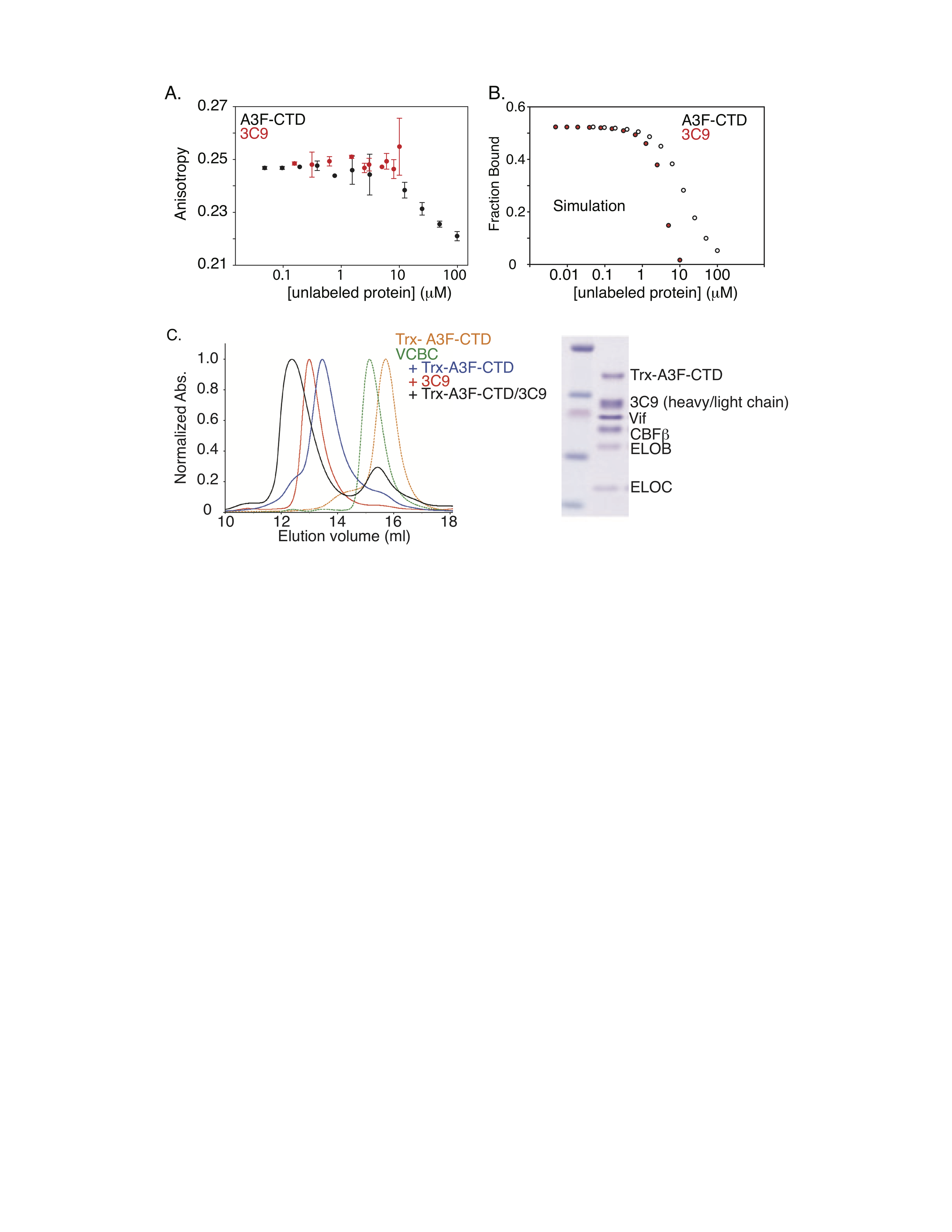

Supplement: S5 Fig — (A) Fluorescence polarization competition assay. Pre-bound fluorescently labeled A3F-CTD with VCBC was titrated with either 3C9 (red) or unlabeled A3F-CTD (black). (B) Simulated competition curves establish that 10 μM of 3C9 and 100 μM of A3F-CTD are sufficient to compete with the labeled A3F-CTD bound to VCBC. (C) SEC elution profile for VCBC/3C9/Trx-A3F-CTD (black), VCBC/3C9 (red), VCBC/Trx-A3F-CTD (blue), VCBC (green), Trx-A3F-CTD (orange). SDS-PAGE gel of peak fraction for VCBC/3C9/Trx-A3F-CTD. (TIF) [file ppat.1006830.s005.tif]

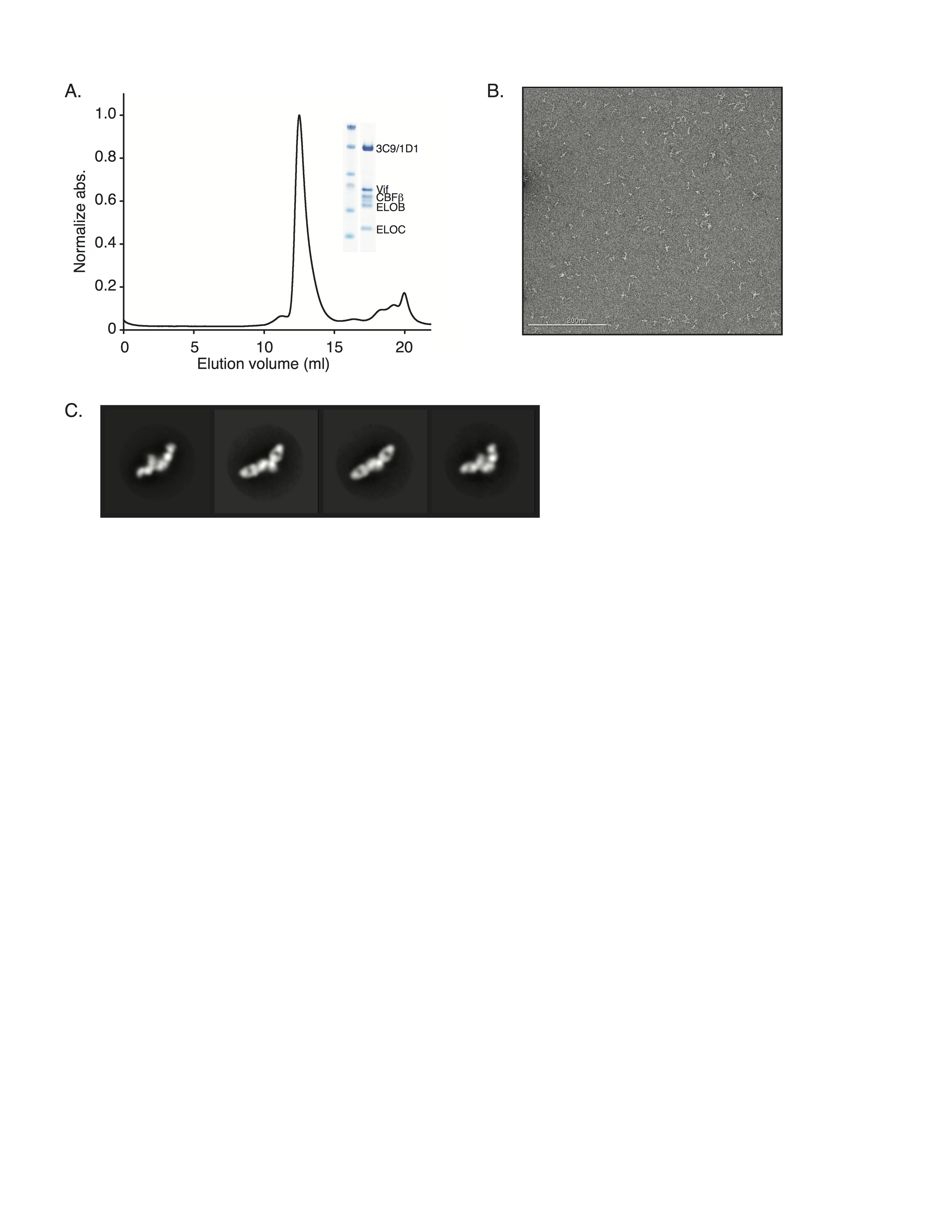

Supplement: S6 Fig — (A) SEC profile and corresponding SDS-PAGE gel for VCBC-3C9-1D1. (B) Representative NSEM micrograph and (C) 2D class averages for VCBC-3C9-1D1. (TIF) [file ppat.1006830.s006.tif]

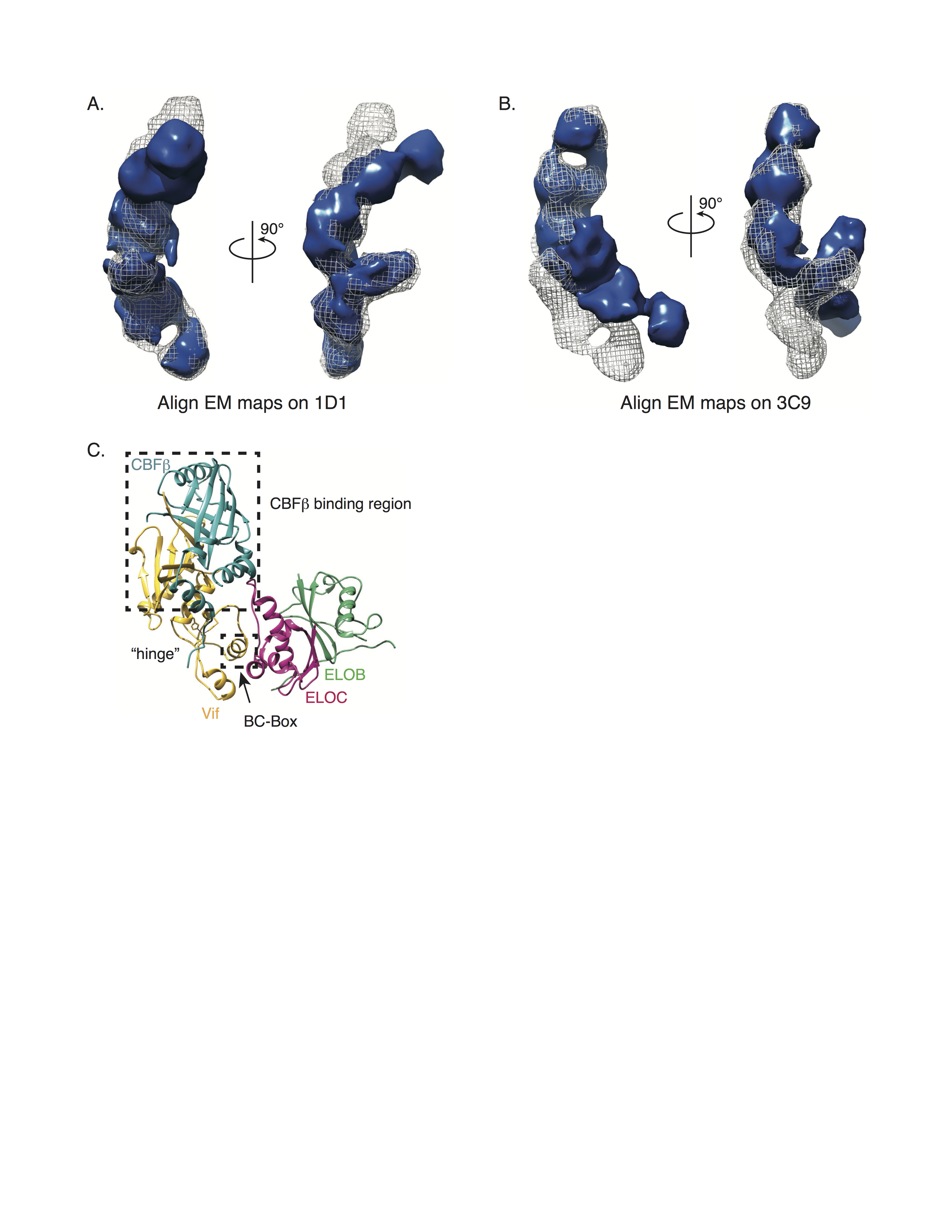

Supplement: S7 Fig — EM maps aligned on (A) 1D1 or on (B) 3C9 reveal that the two conformations arise due to flexibility in VCBC. (C) The CBFβ and ELOC binding regions of Vif are boxed off. Postulated “hinge” connects these two regions and is comprised of the Zn+ binding motif and three interdomain loops. (TIF) [file ppat.1006830.s007.tif]

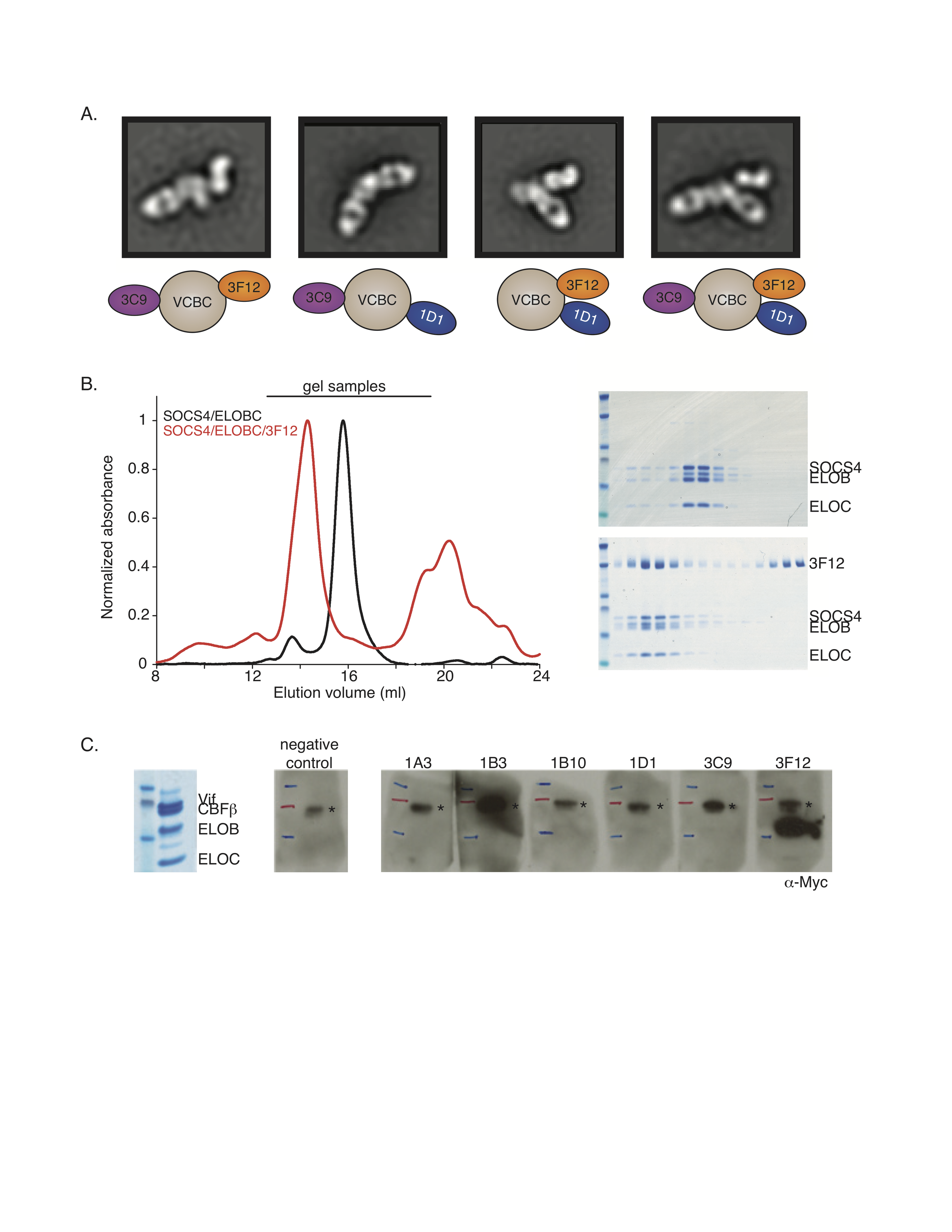

Supplement: S8 Fig — (A) NSEM 2D class averages and corresponding cartoon depictions of VCBC-Fab complexes show that 3F12 and 1D1 bind the same side of VCBC, and that 3C9 binds the opposite side of VCBC from 1D1 and 3F12. (B) SD200 elution profile and corresponding SDS-PAGE gels show that 3F12 is able to form a stable complex with SOCS4/ELOBC. (C) Purified VCBC complex resolved by SDS-PAGE gel. Myc-tagged Fabs were used as a primary antibody to WB for individual VCBC components. 3F12 is able to detect ELOB in a WB. The asterisk indicates a non-specific protein. (TIF) [file ppat.1006830.s008.tif]

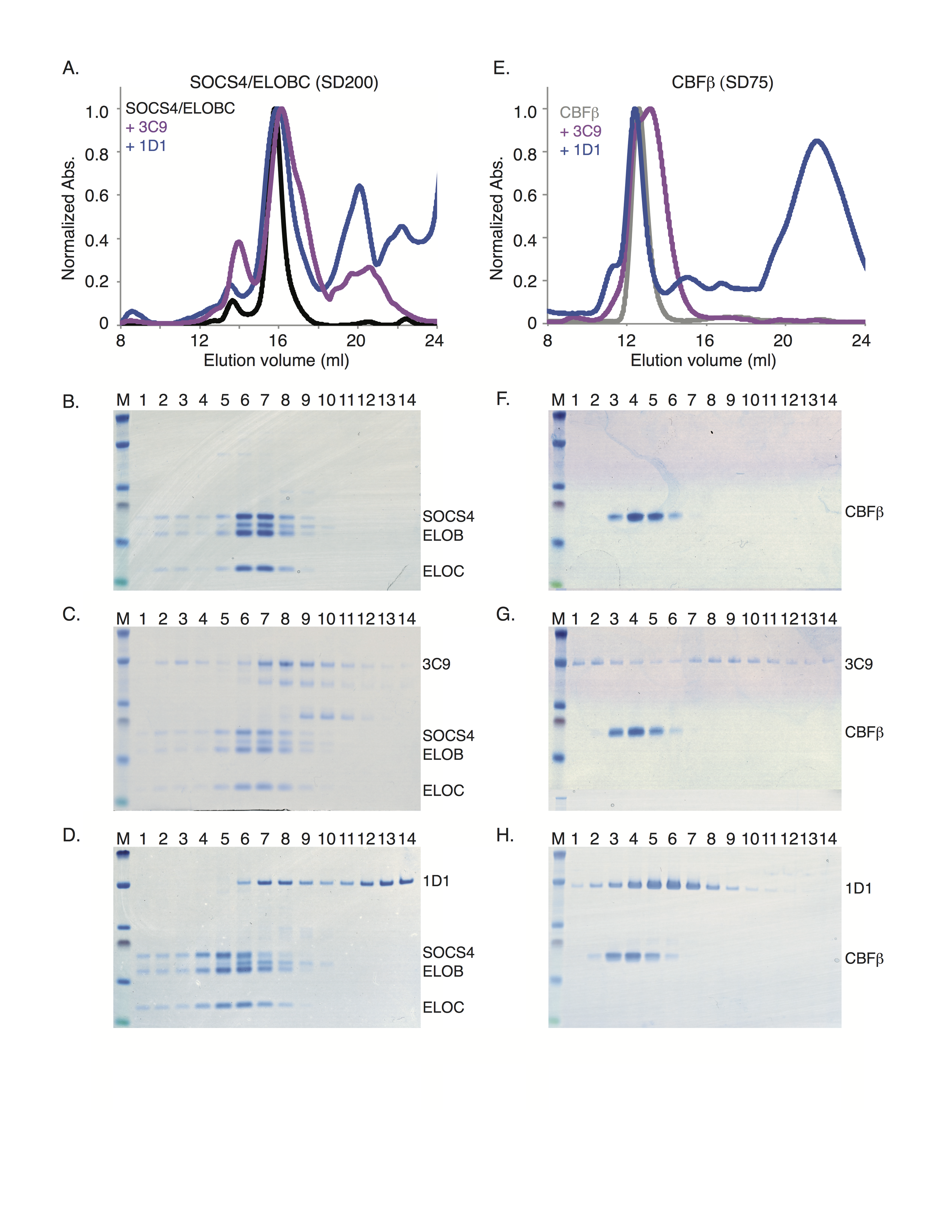

Supplement: S9 Fig — (A) SD200 elution profile and corresponding SDS-PAGE gels for (B) SOCS4/ELOBC and SOCS4/ELOBC in the presence of (C) 3C9 or (D) 1D1. (E) SD75 elution profile and corresponding SDS-PAGE gels for (F) CBFβ and CBFβ in the presence of (G) 3C9 or (H) 1D1. (TIF) [file ppat.1006830.s009.tif]
